# Designation, diligence and drift: understanding laboratory expenditure increases in British Columbia, 1996/97 to 2005/06

**DOI:** 10.1186/1472-6963-12-472

**Published:** 2012-12-21

**Authors:** Saskia N Sivananthan, Sandra Peterson, Ruth Lavergne, Morris L Barer, Kimberlyn M McGrail

**Affiliations:** 1 UBC Centre for Health Services and Policy Research, 201-2206 East Mall, Vancouver, BC, V6T 1Z3, Canada

**Keywords:** Clinical Laboratory Techniques/utilization, Clinical Laboratory Techniques/statistics & numerical data, Guideline care, Diagnostic Techniques and Procedures/economics, Diagnostic Techniques and Procedures/trends, Fee-for-Service Plans/trends, Health Expenditures/trends, Physician's Practice Patterns/trends, Chronic Disease/Condition, British Columbia, Canada

## Abstract

**Background:**

Laboratory testing is one of the fastest growing areas of health services spending in Canada. We examine the extent to which increases in laboratory expenditures might be explained by testing that is consistent with guidelines for the management of chronic conditions, by analyzing fee-for-service physician payment data in British Columbia from 1996/97 and 2005/06.

**Method:**

We used direct standardization to quantify the effect on laboratory expenditures from changes in: fee levels; population growth; population aging; treatment prevalence; expenditure on recommended tests for those conditions; and expenditure on other tests. The chronic conditions selected were those with guidelines containing laboratory recommendations developed by the BC Guidelines and Protocol Advisory Committee: diabetes, hypertension, congestive heart failure, renal failure, liver disease, rheumatoid arthritis, osteoarthritis and dementia.

**Result:**

Laboratory service expenditures increased by $98 million in 2005/06 compared to 1996/97, or 3.6% per year after controlling for population growth and aging. Testing consistent with guideline-recommended care for chronic conditions explained one-third (1.2% per year) of this growth. Changes in treatment prevalence were just as important, contributing 1.5% per year. Hypertension was the most common condition, but renal failure and dementia showed the largest changes in prevalence over time. Changes in other laboratory expenditure including for those without chronic conditions accounted for the remaining 0.9% growth per year.

**Conclusion:**

Increases in treatment prevalence were the largest driver of laboratory cost increases between 1996/97 and 2005/06. There are several possible contributors to increasing treatment prevalence, all of which can be expected to continue to put pressure on health care expenditures.

## Background

Canadians are concerned about the rising cost of health care and particularly about whether the public model is “sustainable” in the face of these cost pressures [1]. Health care was identified as the topic of highest priority for Canadian voters during the 2011 Federal election, as it has been in previous elections [2]. Within the overall landscape of increasing health care costs, expenditures for physician/specialists services in Canada have risen sharply over the past five years, regaining a position as the second largest cost component (exceeded only by acute hospital costs) [3]. Payments for laboratory services, especially for services provided to seniors, have seen particularly rapid increases in British Columbia [4].

The question this raises is what the causes are of laboratory spending increases. Administrative data cannot easily answer the question of health impact associated with this increase in laboratory spending, but it can be used to help diagnose the sources of cost pressure. Repeat testing and technological innovations are likely contributors but are unlikely to explain all of the increases [5-7]. Spending of health care dollars on chronic conditions and the tests associated both with diagnosing and managing those conditions may, on the other hand, help explain the upward trend.

More than half of the Canadian population has at least one chronic condition, and this rises to 81% of community-dwelling seniors [8]. Previous studies have demonstrated that high users of health care services tend to be individuals with (often multiple) chronic conditions [9]. In British Columbia, as in many other provinces, there are guidelines associated with the management of chronic conditions designed to assist physicians in providing consistent, evidence-based care [10]. This includes recommendations for specific laboratory tests to diagnose and/or manage such conditions. It should be noted, the study intent is not an evaluation of adherence to guidelines. Instead, the guidelines are used simply to indicate the consensus on recommended laboratory testing for different chronic conditions. This paper addresses the question: to what extent can increases in laboratory expenditures be explained by testing that is consistent with guidelines for the management of chronic conditions?

Building on previous research, our analyses focus on British Columbia, as no national database exists to allow a more wide-ranging analysis. Our period of analysis is 1996/97 to 2005/06. We describe the extent to which population growth and aging, changes in chronic disease prevalence, and guideline-consistent ordering may explain increased expenditures for laboratory services.

## Methods

### Study population and data sources

Our analyses are of the entire population of British Columbia. The data were accessed through Population Data BC. Data were analyzed at the individual patient level using unique but study-specific codes that do not permit personal identification of either patients or physicians. Permission for data access was provided by the BC Ministry of Health. Ethics approval for this research was granted by the University of British Columbia Behavioural Research Ethics Board.

We accessed the following files: 1) a central demographics file for 1996/97 and 2005/06 providing information on age and sex of individuals and denominator information for the analyses; and 2) the fee-for-service payment files for 1995/96-1996/97 and 2004/05-2005/06 as a two year period is required to confirm a diagnosis. The fee-for-service data include the date of each visit, total amount paid, a unique study-specific physician identification number as well as a study-specific patient identification number, the physician specialty code, the diagnostic (ICD9) code most responsible for the visit, a fee item code which is a code used to identify each service provided by a practitioner, and a service code, which is a grouping of the fee items that indicates the type of services rendered by a practitioner. We removed the effects of fee changes over this period by valuing services provided in all years at the fee levels in effect on April 1, 2005/06, yielding fee-adjusted expenditures [4].

### Classifying chronic conditions

The chronic conditions selected for this study were those for which specific guidelines developed by the BC Guidelines and Protocol Advisory Committee for the Medical Services Commission containing laboratory recommendations which came into place before 2006 [10]. These were diabetes, hypertension, congestive heart failure, renal failure, liver disease, rheumatoid arthritis, osteoarthritis and dementia. Cancer was excluded because the spectrum of diseases encompassed by the term resulted in patient profiles with more widely varying patterns of use than those with the other chronic conditions. There are other chronic conditions not included in this analysis that will have associated laboratory tests. Increases in testing for those conditions will be captured as general increases, since our research question is focused on testing related to guidelines.

Chronic conditions were identified using the first 3 digits only of the International Classification of Disease (ICD-9) diagnosis codes from records of fee-for-service payments to general physicians, medical specialists and surgical specialists. Individuals were counted as having a chronic condition if they had at least two records showing a diagnosis for the same condition over a two-year period. This approach to determining treatment prevalence is consistent with prior research using administrative data to identify individuals with chronic conditions [8,11,12]. Treatment prevalence is used here to acknowledge the limitations of administrative data in identifying the prevalence of disease. While administrative data have been shown to be quite valid in for this purpose [12], we are only counting people as having disease if they received services from a physician who recorded relevant diagnoses on a billing record. Individuals with one record showing a diagnosis of a chronic condition were counted in a ‘potential’ category, as the diagnosis may have been recorded as something to be ruled out rather than being definitive. Payments from 1995/96 – 1996/97 were used to identify chronic conditions for the 1996/97 study population, and 2004/05-2005/06 payments were used to identify chronic conditions for the 2005/06 study population.

We summarized the information on chronic conditions and created five mutually exclusive groups to reflect an overall chronic disease profile for each individual: ‘No Guideline-Related Chronic Condition’, ‘Potential Chronic Condition’, ‘One’, ‘Two’ or ‘Three plus Chronic Conditions’. Individuals who had no record of a chronic condition diagnosis during the two-year period were classified in the first category. Those who received only one record showing a diagnosis for one or more conditions were classified into the “Potential Chronic Condition” group. Those with two or more records showing diagnoses for one condition (irrespective of “potential” conditions) were counted in the “one Chronic Condition” group, and so on. Individuals in these profile groups reflect categorization based on the chronic diseases with guidelines identified earlier, as per our research question, but may still have other chronic conditions outside of the scope of the study.

### Laboratory testing

Medical laboratory testing in British Columbia is provided by community-based and hospital-based laboratories. This study included all laboratory tests paid for through the Medical Services Plan (MSP) of BC., i.e. not through hospital global budgets. The latter generally include only inpatient laboratory services, so this was not a major limitation.

Payments for laboratory testing were identified in the MSP data using service codes, regardless of the type of practitioner that billed for the item. A cluster of laboratory tests was created for each of the eight chronic conditions examined, based on the test recommendations made by the BC Guidelines and Protocol Advisory Committee (see Additional file 1 for details). All other tests were classified as not attributable to the chronic conditions we were examining. The primary base fee is an administrative cost (similar to a pharmacy dispensing fee) that is applicable under specified criteria to certain panel tests performed within the same facility. It could not be allocated to specific laboratory tests but consisted of a large portion of expenditure and therefore was treated separately. Total payments and payments for test subsets by chronic condition were then summed for each person in the study population. Some tests are associated with more than one chronic condition. Allocating individuals to the mutually exclusive chronic disease profile groups and summing separately avoided double-counting.

### Analysis

We approached the analysis using the following expression:

(1) $$\begin{array}{rl} \text{Total}\;\text{Lab}\;\text{Exp}= & N_{c}\left(\frac{\mathrm{\$Test}s_{r}}{N_{c}}+\frac{\mathrm{\$Test}s_{o}}{N_{c}}\right)\\ & +N_{\mathrm{nc}}\left(\frac{\mathrm{\$Test}s_{o}}{N_{c}}\right) \end{array}$$

Where Total Lab Exp is the total expenditure on laboratory testing in a given year, N_c_ is the number of people with chronic conditions, N_nc_ is the number of people without guideline-related chronic conditions, $Tests_r_ is the expenditure on recommended laboratory tests for chronic conditions and $Tests_o_ is the expenditure on other laboratory tests. Using this expression we can see that after controlling for changes in fees, overall population growth and population aging, changes in laboratory expenditures will come from changes in the number of people with chronic conditions (the treatment prevalence), changes in the expenditure on recommended tests for each person, or changes in per person expenditure on other tests. We isolate these three components in our analyses using direct standardization.

## Results

### The cost of utilization

Between 1996/97 and 2005/06, real (constant dollar) health care services expenditures rose by 25.4% (Table 1). Laboratory service expenditures rose much faster -- $98.0 million, or 58.7%, an average of 5.3% per year. Accounting for the growth of the BC population during the study period, per capita laboratory costs increased from $41.80 in 1996/97 to $60.40 in 2005/06, still an extraordinary 44.7% growth over the nine years. Aging of the population contributed 5.3% (about 0.6% per year), leaving 37.4% of the per capita growth attributable to changes in prevalence and changes in laboratory testing intensity.

**Table 1:** **Dynamics of laboratory expenditures in British Columbia**, **1996**/**97 and 2005**/**06**

			**%Change**	
	**1996**/**97**	**2005**/**06**	**Overall**	**Average annual**
Total health care services expenditures (constant $)	1,594,591,397	1,998,842,372	25.4%	2.5%
Per capita health care services expenditures	399	455	14.3%	1.5%
Total lab expenditures (constant $)	166,914,987	264,904,987	58.7%	5.3%
BC Population	3,999,520	4,383,445	9.7%	1.0%
Per capita lab expenditures	41.8	60.4	44.7%	4.2%
			**%Growth attributable**	
Growth attributable to change in age structure			5.3%	0.6%
Growth attributable to other changes			37.4%	3.6%

**Note**: All numbers reflect age-standardization within broad age bands. We used a direct standardization approach, standardizing 1996/97 utilization rates to the 2005/06 population.

**Notes for reading Tables**1 &2: Rows are only additive for "expenditures per capita". For the other terms of the equation, the denominators differ by column and so it is impossible to add the values.

### Demography, treatment prevalence and co-morbidities

In examining chronic condition guideline-consistent laboratory test expenditures, one factor that may contribute to overall expenditure growth is any population-level changes in the treatment prevalence of the identified conditions. If age-specific prevalence is increasing, then, laboratory-related expenditures would increase faster than what we would expect as a result of population aging alone.

**Figure 1:**
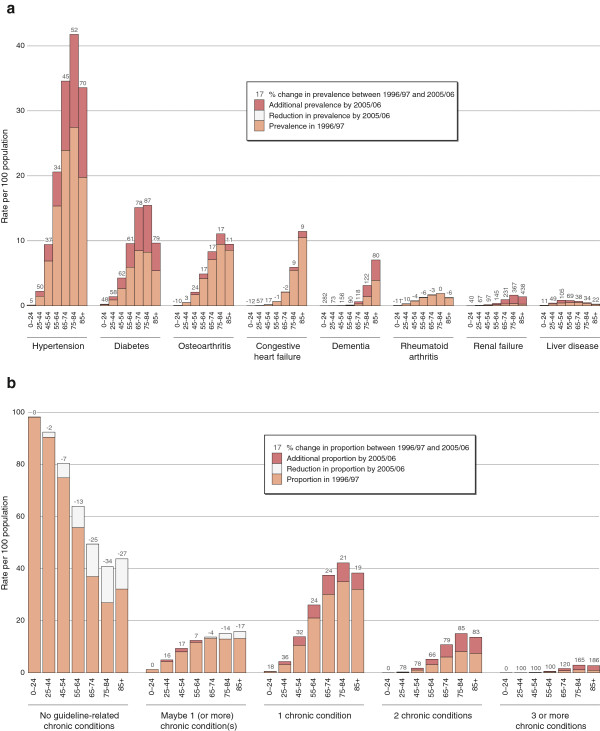
**a:Change in treatment prevalence of selected chronic conditions between 1996**/**97 and 2005**/**06**, **by age group.b**: Change in proportion of individuals in different chronic disease categories between 1996/97 and 2005/06.

The treatment prevalence increased for seven out of eight of the chronic diseases examined in this study. Hypertension was the most common condition, affecting 6.0% of the population in 1996/97 and 9.9% in 2005/06. However, the most rapid increases in treatment prevalence were found for renal failure and dementia -- 227.0% and 145.6% increases respectively (Figure 1A) -- albeit on a much smaller starting population than for hypertension.

Figure 1B then illustrates the rapid change in the proportion of individuals in the different chronic disease categories between 1996/97 and 2005/06. An increasing number of individuals were classified as having one or more chronic conditions in 2005/06. The largest percentage increase, which reached triple digits in all age categories except for the youngest age group of 0–24 years, was seen for individuals classified as having three or more chronic conditions.

### Intensity of service use

**Figure 2:**
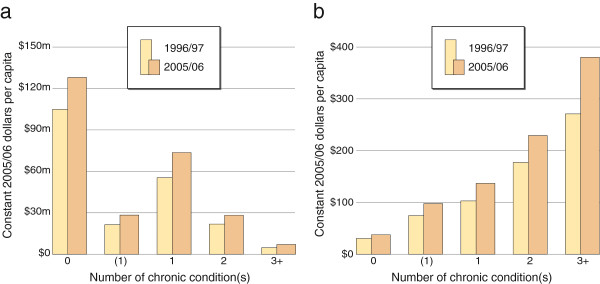
**a:Total laboratory expenditure on individuals in different chronic disease categories**, **age standardized**, **between 1996**/**97 and 2005**/**06.** The total laboratory expenditure on individuals with no guideline-related chronic disease, maybe one chronic disease, one, two and three or more chronic diseases in 1996/97 and 2005/06. **b**: Per capita laboratory expenditures on individuals in different chronic disease categories, age standardized, between 1996/97 and 2005/06. The per capita laboratory expenditures on individuals with no guideline-related chronic disease, maybe one chronic disease, one, two and three or more chronic diseases in 1996/97 and 2005/06.

Total expenditures and expenditures per capita increased over time for all chronic condition groups (Figure 2A). At both time points, the highest total expenditure was on those without chronic conditions, while the lowest total expenditure was on individuals with three or more co-morbidities. In fact, the group with the biggest percent increase in expenditure between 1996/97 and 2005/06 was also the no guideline-related chronic condition category.

The intensity of service use, as demonstrated by per capita costs, tells a more nuanced story. As expected, intensity of service use was higher among those with chronic conditions, and increased both with the number of co-morbidities and over time (Figure 2B). The pattern of care for individuals classified as having three or more chronic conditions is of particular note because this category had the most rapid change in expenditure over the period.

### Chronic condition guideline-consistent laboratory expenditures

Total expenditures for chronic condition guideline-consistent laboratory tests increased from $9.9 million to $28.6 million, a 188.2% change (Table 2). Total expenditures for all other laboratory tests increased from $138.0 million to $207.5 million, a still substantial but much smaller 50.3% increase. So while guideline-consistent laboratory tests showed a much steeper increase over time, the other laboratory tests continued to represent the more significant share of total expenditure, even at the end of the period. The 75–84 age group had the highest per capita expenditure for guideline-consistent laboratory tests as well as per capita expenditures for other laboratory tests.

**Table 2:** **Disaggregation of laboratory expenditures in British Columbia**, **1996**/**07 and 2005**/**06**

									**% Change**			
					**1996**/**97**		**2005**/**06**		**Overall**		**Average annual**	
Total expenditure for guideline-consistent laboratory tests					9,929,315		28,615,178					
Total expenditure for other laboratory tests					138,034,815		207,496,629		50.3%		4.6%	
**Guideline**-**consistent lab expenditures by age group**	**Per capita guideline consistent laboratory tests**		**$change**	**%change**	**Per capita other laboratory tests**		**$change**	**%change**	**Per capita base fee**		**$change**	**%change**
	**1996**/**97**	**2005**/**06**			**1996**/**97**	**2005**/**06**			**1996**/**97**	**2005**/**06**		
0 to 24	0.1	0.2	0.1	64.0%	17.3	19.2	1.9	10.9%	1.1	1.3	0.2	16.5%
25 to 44	0.7	1.5	0.8	106.3%	36.3	45.0	8.7	24.0%	3.2	3.8	0.6	20.1%
45 to 54	2.8	5.8	3.0	109.7%	38.8	52.4	13.5	34.9%	5.9	7.6	1.6	27.7%
55 to 64	6.5	13.6	7.1	108.8%	46.1	65.7	19.5	42.3%	9.3	11.8	2.4	26.3%
65 to 74	9.9	24.0	14.0	141.0%	58.5	84.1	25.6	43.7%	12.9	16.7	3.8	29.5%
75 to 84	10.3	27.8	17.5	170.0%	65.8	99.2	33.5	50.9%	14.5	19.4	4.9	33.8%
85 plus	8.2	20.0	11.9	145.4%	55.0	82.5	27.5	49.9%	12.7	16.3	3.5	27.8%
							**%Growth attributable**					
							**Overall**			**Average annual**		
Growth attributable to other changes (from Table 1)							37.4%			3.6%		
Growth attributable to changes in prevalence							13.9%			1.5%		
Growth attributable to changes in utilization							20.7%			2.1%		
Growth attributable to changes in guideline-consistent laboratory testing							11.3%			1.2%		
Growth attributable to changes in other laboratory testing							8.5%			0.9%		

### Summing up the effects

As shown in Table 1, 37.4% (3.6% average annual increase) of growth in laboratory expenditures between 1996/9/7 and 2005/06 was attributable to changes other than aging and population growth. Of this, 13.9% (1.5% per year) was due to changes in treatment prevalence. Disaggregating the remaining 20.7% (2.1%) further, chronic condition guideline-consistent laboratory tests accounted for 11.3% (1.2%) of the growth and changes in other laboratory tests 8.5% (0.9%).

## Discussion

Spending on laboratory testing in BC increased 58.7% between 1996/97 and 2005/06. Many factors, including changes in technology, repeat testing and inappropriate utilization have been proposed as potential factors contributing to the increase in laboratory utilization [7]. One additional explanation for expenditure increases is increased testing consistent with medical practice guidelines for diagnosing and monitoring patients with chronic conditions. We find that there was, indeed such an increase between 1996/97 and 2005/06, but that this explains only about one-third of the total increase, net of population growth and aging. Changes in treatment prevalence were equally important, and unexplained increases in other laboratory tests for people with and without the specific chronic conditions of interest were a large contributing factor.

Several changes in the clinical definition and screening of chronic conditions occurred between 1996/97 and 2005/06. For example, the clinical definition of diabetes shifted from a fasting plasma glucose level of greater than or equal to 7.8 to 7.0 mmol/L [13,14]. For renal disease, serum creatinine levels of 176.8 μmol/L in men (eGFR of ~40 mL/min/1.73 m^2^ for a 40 year old man) resulting in referral to a renal team for dialysis assessment, changed to every patient with urine abnormalities and an eGFR <90 mL/min/1.73 m^2^ receiving immediate further assessment and management [15,16]. Similarly, the recommended screening guidelines for Type 2 diabetes shifted to begin at age 41 instead of age 46 [13,14]. There were no major changes in clinical definitions or screening guidelines for osteoarthritis or rheumatoid arthritis and for those conditions we see relatively stable prevalence across age groups over time^11^. These changes are only two factors that may be driving the increase in treatment prevalence for these chronic conditions, but they are surely significant ones. Once people are diagnosed with a condition, it should be no surprise that associated health care, and particularly guideline-consistent expenditures will follow.

There has also been a trend during this period toward diagnosing “pre-disease” states as part of screening guidelines, and a growing demand from patients for tests of their choosing even if the physician may not consider them beneficial. As previously mentioned, the first screen for diabetes is now recommended at earlier ages regardless of risk factors with a diagnosis of “impaired fasting glucose” as a “pre-diabetic” state at fasting plasma glucose levels of 6.1 mmol/L. These individuals being flagged as “at risk” subsequently require follow up tests until the disease manifests itself [14]. So recommendations for increased population screening at ever earlier ages and the lower threshold for these pre-disease "conditions" that then leads to increased monitoring of individuals with no disease with testing could be another driver of the increase in other laboratory tests [14,17,18].

Much has been made of the aging population and its impact on the healthcare system. However, only 5.3% of the growth in laboratory expenditure over this period was actually attributable to a change in the age structure. More is spent on the elderly and this trend has continued between 1996/97 to 2005/06. However, the impact of an increasing treatment prevalence of age-specific chronic conditions, and particularly of an increasing age-specific prevalence of patients with multiple co-morbidities, is far more important.

There are some limitations to this study. The fee-for-service physician data do not include information on services paid for through alternative payment arrangements such as salaries, sessional payments, or contractual arrangements. This does not affect the laboratory payments, which are all by fee-for-service for patients outside of acute care, but may affect our classification of these individuals into the chronic disease categories. In addition, our analysis of chronic conditions was not exhaustive, but rather focused only on those conditions that became the subject of incentive programs for primary care in BC. The “other” laboratory tests, while not guideline-recommended for these specific chronic conditions, may still be appropriate for other conditions. Therefore individuals with other potentially prevalent chronic conditions that were outside the scope of the conditions selected for this study would be classified as ‘no guideline-related chronic condition’, which limits our interpretation of people in this category.

## Conclusion

Laboratory testing is one of the fastest growing areas of physician service provision. In British Columbia, there was just under $100 million in “new” laboratory expenditures in 2005/06 compared to a 1996/97 base of $167 million. This amounts to a 5.3% average annual increase in expenditure, or 3.6% per year after removing the effects of population growth and aging. Our primary research question was the extent to which diligence – testing consistent with guideline-indicated care – could explain this increase. The answer is that about one-third of the increase, or about 1.2% per year, can be attributed to changes in guideline-consistent test ordering. Surprisingly important are designation – the increased likelihood of being diagnosed with a chronic condition – at 1.5% per year, and drift – the general increase in laboratory expenditures, including expenditures for people without these guideline-related chronic conditions - at 0.9% per year. This suggests that future research might productively focus on the last of these contributing components, with an emphasis on the extent to which the increase in designation and the general increase in testing affects diagnosis or subsequent treatment.

## Abbreviations

BC: British Columbia; MSP: Medical Service Plan; ICD: International Classification of Diseases; eGFR: Estimated glomerular filtration rate.

## Competing interests

The authors declare that they have no competing interests.

## Authors’ contributions

All authors contributed to the interpretation of the data. KM and MB conceived and developed the initial design of the study and acquired the data. SS managed the study, further developing the design, conducted the analysis and interpretation of the data. SP and RL contributed significantly to the analysis and interpretation. SS wrote the first draft and managed subsequent drafts with revisions from all other authors. All authors read and approved the final manuscript.

## Pre-publication history

The pre-publication history for this paper can be accessed here:

http://www.biomedcentral.com/1472-6963/12/472/prepub

## Supplementary Material

Additional file 1 BC Guidelines and Protocol Advisory Committee recommended laboratory tests attributed to each of the eight chronic conditions examined. Click here for file
