# Supplementary material for: Designation, diligence and drift: understanding laboratory expenditure increases in British Columbia, 1996/97 to 2005/06
Source: BMC Health Serv Res. 2012 Dec 21;12:472. doi: 10.1186/1472-6963-12-472 (PMC3542022; doi:10.1186/1472-6963-12-472)
Supplement: Additional file 1 — BC Guidelines and Protocol Advisory Committee recommended laboratory tests attributed to each of the eight chronic conditions examined. [file 1472-6963-12-472-S1.doc]

**Additional file 1 BC Guidelines and Protocol Advisory Committee recommended laboratory tests attributed to each of the eight chronic conditions examined.**

| **CHRONIC CONDITION** | **RECOMMENDED LABATORY TEST** | |
| --- | --- | --- |
| **Diabetes** | - Plasma glucose - Fasting plasma glucose | - Glycosylated haemoglobin (A1C) - Fasting lipid profile (high density, low density cholesterol, triglycerides, total cholesterol) |
| **Hypertension** | - Urinalysis - Blood chemistry (potassium, sodium, creatinine/estimated glomerular filtration rate) - Fasting blood glucose | - Fasting total cholesterol - Lipids profile - Microalbuminuria (albumin/creatinine ratio) |
| **Congestive Heart Failure** | - Complete blood count - Serum electrolytes - Creatinine - Estimated glomerular filtration rate (eGFR) - Urinalysis - Microalbuminuria | - Fasting blood glucose - Fasting lipid profile - Aspartate Aminotransferase (AST) - Albumin - Thyroid-stimulating hormone |
| **Renal Failure** | - Estimated glomerular filtration rate (eGFR) - Macroscopic urinalysis | - Microscopic urinanalysis - Albumin/creatinine ratio |
| **Liver Disease** | - Alanine aminotransferase (ALT) - Alkaline phosphatase (ALP) - Aspartate Aminotransferase (AST) - Gamma-glutamyltransferase (GGT/GTP) - Serum albumin - Prothrombin Time | - Auto-antibodies - Copper - Iron studies - Alpha-feto protein (AFP) - Specific viral markers |
| **Rheumatoid Arthritis** | - Erythrocyte Sedimentation Rate (ESR) or C-Reactive Protein (CRP) | - Rheumatoid Factor - Antinuclear Antibody (ANA) |
| **Osteoarthritis** | - Complete blood count - Haemoglobin - Creatinine | - Aspartate Aminotransferase or Alanine aminotransferase (AST or ALT) |
| **Dementia** | - Complete blood count - Serum electrolytes - Serum calcium | - Serum glucose - Thyroid Stimulating Hormone - B12 |
